# Divergent roles of Hsp70 chaperones in orthoflavivirus protein secretion and virion formation

**DOI:** 10.1038/s44298-026-00175-8

**Published:** 2026-02-02

**Authors:** Lea Blank, Christin Lorenz, Imke Steffen

**Affiliations:** 1 Institute of Biochemistry, University of Veterinary Medicine, Hannover, Germany; 2 Research Center for Emerging Infections and Zoonoses, University of Veterinary Medicine, Hannover, Germany; 3 Department of Microbiology, Immunology and Pathology, College of Veterinary Medicine and Biomedical Sciences, Colorado State University, Fort Collins, CO USA

**Keywords:** Biochemistry, Microbiology

## Abstract

Orthoflaviviruses, such as tick-borne encephalitis virus (TBEV) and West Nile virus (WNV), can cause severe neurological disease and remain without specific antiviral treatments. We found that orthoflavivirus envelope (E) and non-structural protein 1 (NS1) interact with heat shock protein 70 (Hsp70) chaperones, key regulators of protein homeostasis and existing cancer drug targets. We examined how Hsp70 and endoplasmic reticulum–resident BiP contribute to viral protein secretion and infectivity of tick and mosquito-borne orthoflaviviruses. Targeting the Hsp70 nucleotide-binding domain with small-molecule inhibitor YM-1 significantly reduced infectivity of multiple orthoflaviviruses, while substrate-binding domain inhibitor PES-Cl specifically impaired NS1 secretion of tick-borne orthoflaviviruses. Protein degradation inhibitors restored NS1 expression in BiP-deficient cells but failed to rescue NS1 secretion. These data indicate that while BiP is essential for secretion of tick-borne orthoflavivirus NS1, it is not required for infectivity. The antiviral effect of YM-1 likely reflects inhibition of other chaperones or additional cellular targets.

## Introduction

Vector-borne viral infections are an increasing global health concern, with the incidence of arthropod-transmitted diseases rising due to ecological and climate-driven changes. *Orthoflaviviruses*, a genus within the *Flaviviridae* family, are transmitted by mosquitoes or ticks and include medically important pathogens, such as West Nile virus (*Orthoflavivirus nilense*, WNV) and tick-borne encephalitis virus (*Orthoflavivirus encephalitidis*, TBEV), which can cause severe disease in humans and animals, including West Nile fever and tick-borne encephalitis^1–3^. Despite their growing impact, no specific antiviral treatments are available, highlighting the urgent need for further research into these pathogens^4–7^.

Orthoflaviviruses are small, enveloped viruses with a positive-sense single-stranded RNA genome. The RNA genome encodes a single open reading frame that is translated into a single polyprotein at the membrane of the host cell endoplasmic reticulum (ER). The polyprotein is cleaved by both viral and host proteases into three structural proteins—capsid (C), pre-membrane (prM), and envelope (E)—and seven non-structural proteins (NS1, NS2A, NS2B, NS3, NS4A, NS4B, NS5)^8^. During translation, prM, E, and NS1 extend into the ER lumen, while the remaining proteins are embedded in the ER membrane or located on the cytoplasmic face of the ER membrane^9^. The E protein is a membrane-bound glycoprotein essential for viral entry. It mediates attachment to host cell receptors, facilitates endocytic uptake of the virus, and mediates fusion of the viral and endosomal membranes^10^. As the major viral antigen, E is the primary target of neutralizing antibodies and a key focus for vaccine and antiviral drug development. NS1, another glycoprotein, exists in multiple oligomeric forms that serve distinct functions. Following translation, NS1 is synthesized as a soluble monomer in the ER lumen, where post-translational modifications enable its dimerization and membrane association^11^. The membrane-bound NS1 dimer is essential for viral replication, acting as a cofactor within the replication complex and colocalizing with double-stranded RNA^12,13^. Some NS1 proteins are further processed through the secretory pathway, undergoing complex glycosylation in the Golgi before being secreted as tetramers or hexamers^14–16^. In its soluble form, NS1 can be detected in serum and other body fluids, including cerebrospinal fluid^17–20^. Additionally, NS1 plays immunomodulatory roles, such as complement inhibition and Toll-like receptor activation^21,22^, and has been implicated in viral pathogenesis, contributing to neuroinvasiveness and vascular leakage^23,24^.

Like other RNA viruses, orthoflaviviruses rely on the host ER and its biosynthetic machinery to facilitate their replication and virion formation. They extensively remodel ER membranes to form vesicle packets that provide a dedicated environment for viral genome replication and protein synthesis^25^. These membrane reorganizations, coupled with the accumulation of viral proteins in the host ER, impose significant stress on the organelle, triggering the unfolded protein response (UPR)^26^. The UPR is a cellular stress response that restores ER homeostasis by increasing the ER protein-folding capacity, transiently reducing host mRNA translation, and enhancing ER-associated degradation (ERAD) of misfolded proteins^27^. Three key ER stress sensors - activating transcription factor 6 (ATF6), inositol-requiring enzyme 1 (IRE1), and PKR-like ER kinase (PERK) - regulate this pathway^28^. Under normal conditions, these sensors are held in an inactive state by transient interactions with the ER-resident chaperone BiP/GRP78 (*HSPA5* gene). BiP, a member of the heat shock protein 70 (Hsp70) family, is a central regulator of ER protein homeostasis^29^. Like other Hsp70 chaperones, BiP consists of two functional domains: an N-terminal nucleotide-binding domain and a C-terminal substrate-binding domain^30^. In response to ER stress, BiP dissociates from the three UPR sensors to bind exposed hydrophobic regions of unfolded proteins, thereby activating the stress response and facilitating proper protein folding^31^. Orthoflaviviruses are known to manipulate UPR signaling pathways and exploit host chaperones to support viral replication^26^. Infections with various orthoflaviviruses, including tick-borne encephalitis virus (TBEV) and West Nile virus (WNV), lead to a strong upregulation of UPR signaling^32–34^. Our previous work demonstrated that TBEV and Langat virus (*Orthoflavivirus langat*ense, LGTV) infection activate the UPR, with BiP being among the most strongly induced chaperones^34^. However, whether BiP directly interacts with newly synthesized viral proteins in the ER, and how this interaction might contribute to viral replication, remains unclear. Denolly et al. previously identified BiP as a key interactor of DENV NS1, with its expression upregulated during DENV infection through the UPR^35^. They could show that BiP is required for proper DENV NS1 folding and secretion, which is disrupted when UPR activation is impaired. Similarly, the prototype member of the Hsp70 family, Hsp70 (HSPA1), though localized predominantly in the cytosol, has previously been shown to be required for DENV replication^36^. Depletion of cytosolic Hsp70 isoforms and overexpression of dominant-negative Hsp70 reduced DENV propagation, suggesting proviral roles for several different members of this chaperone family.

This study sheds light on the specific roles of Hsp70 family chaperones, particularly BiP (HSPA5) and Hsp70 (HSPA1), in the life cycle of orthoflaviviruses, including both tick- and mosquito-borne species. We hypothesized that disrupting chaperone interactions with viral proteins will affect distinct stages of the orthoflavivirus life cycle. By examining these interactions and evaluating their functional relevance during infection, we sought to identify host chaperone pathways that could serve as potential targets for antiviral intervention.

## Results

### ER chaperones BiP and Hsp70 form complexes with the E proteins of different orthoflavivirus species

**Fig. 1:**
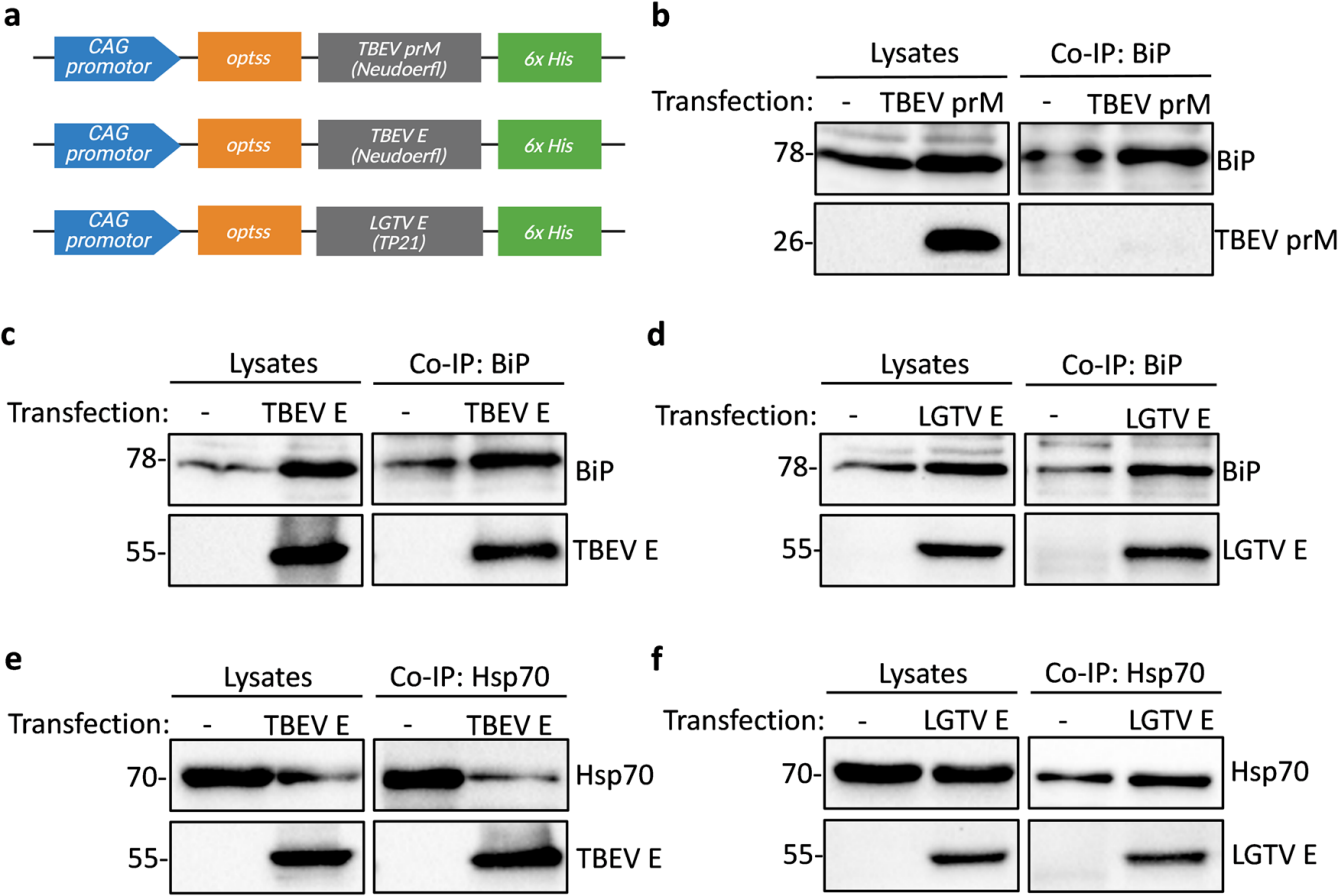
ER chaperones BiP and Hsp70 form complexes with the overexpressed E proteins of tick-borne orthoflavivirus species. **a** Schematic representation of the expression constructs for different viral glycoproteins that include artificial optimized signal sequences (optss) to ensure proper insertion of the proteins into the ER membrane. **b**–**d** Overexpression of TBEV prM (**b**), TBEV E (**c**), and LGTV E (**d**) proteins in HEK293T cells followed by immunoprecipitation of BiP and western blot detection of BiP and the respective viral proteins in lysates and precipitated samples. BiP was detected with an anti-BiP antibody, TBEV prM with anti-LGTV prM and TBEV and LGTV E with anti-LGTV E antibody. Cells were transfected with empty vector as a negative control (-). Representative blots of at least three independent replicates. **e**, **f** Overexpression of TBEV E (**e**), and LGTV E (**f**) proteins in HEK293T cells followed by immunoprecipitation of Hsp70 and western blot detection of Hsp70 and the respective viral proteins in lysates and precipitated samples. Hsp70 was detected with an anti-Hsp70 antibody and TBEV and LGTV E with anti-LGTV E antibody. Representative blots of at least three independent replicates are shown. Input lysates are shown as controls for co-immunoprecipitations.

BiP is a key regulator of the unfolded protein response, but its role in orthoflavivirus protein synthesis is less defined. To investigate whether BiP interacts directly with viral proteins of tick- and mosquito-borne orthoflaviviruses, we tested whether BiP associates with the ER-luminal prM and E proteins of tick-borne orthoflaviviruses in an overexpression system in HEK293T cells that express the prM and E proteins of TBEV Neudoerfl and the E protein of LGTV TP21. An optimized signal sequence^37^ was added upstream of the protein-coding sequence to all constructs to ensure proper co-translational translocation of the prM and E proteins into the ER lumen (Fig. 1a). Co-immunoprecipitation (co-IP) using a BiP-specific antibody revealed that BiP did not interact with TBEV prM (Fig. 1b), but formed complexes with the E proteins of both LGTV and TBEV (Fig. 1c, d). Lack of interaction between BiP and TBEV prM is consistent with the notion that BiP preferentially engages misfolded or incompletely folded proteins and suggests that the smaller prM protein either folds efficiently in the ER or does not expose hydrophobic patches recognized by BiP. Given that BiP belongs to the Hsp70 family, which includes other functionally related chaperones, we also tested whether Hsp70 interacts with the two orthoflavivirus E proteins. Co-IP confirmed that Hsp70 similarly associates with overexpressed LGTV and TBEV E proteins (Fig. 1e, f). Because co-immunoprecipitation is a qualitative assay, we present these data as evidence of interaction rather than a quantitative comparison of band intensities, which can be confounded by technical factors, such as antibody concentration and buffer conditions.

**Fig. 2:**
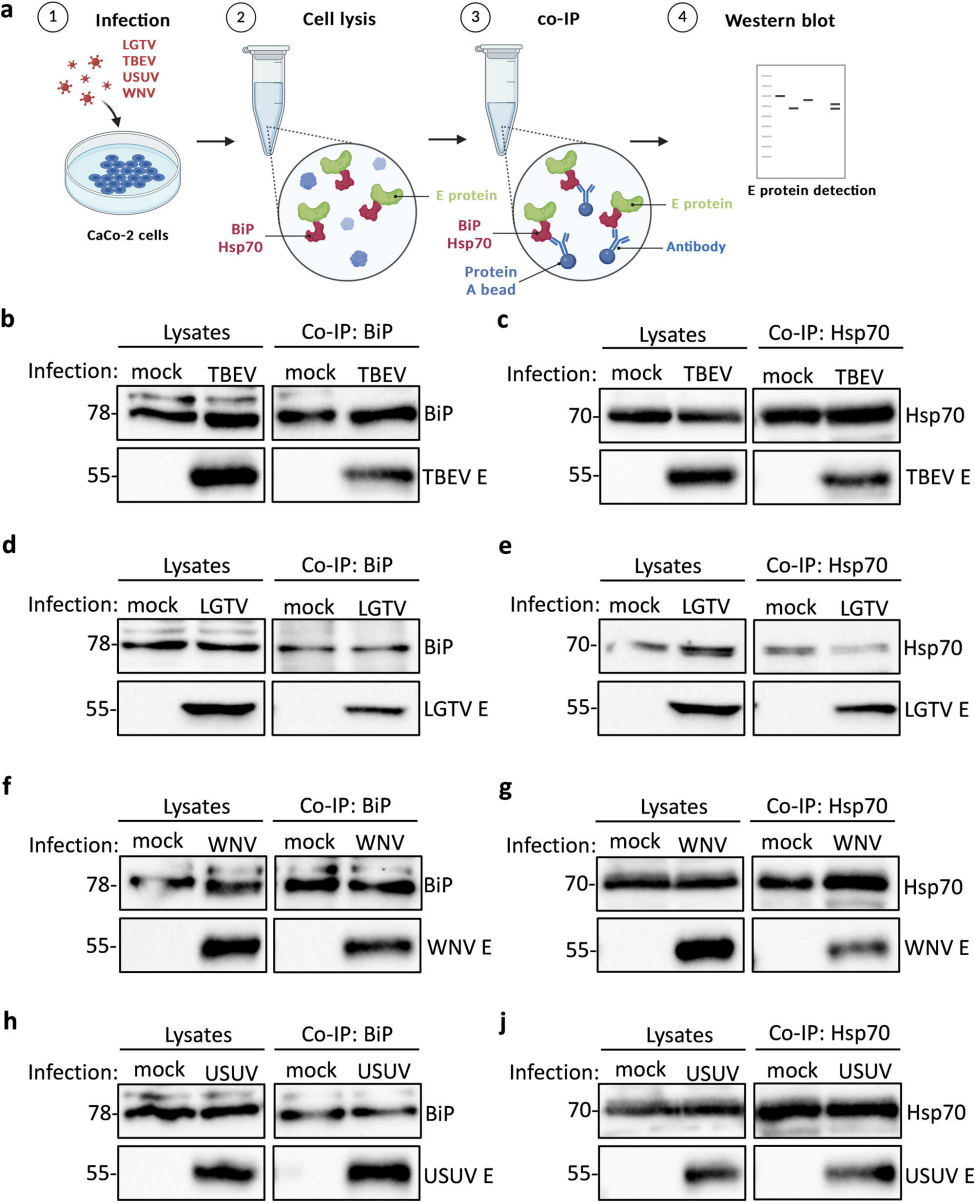
ER chaperones BiP and Hsp70 form complexes with orthoflavivirus E proteins during infection. **a** Schematic representation of the workflow for infection/co-immunoprecipitation experiments. **b–j** CaCo-2 cell infection with TBEV (**b**, **c**), LGTV (**d**, **e**), WNV (**f**, **g**), or USUV (**h**, **j**) (MOI 0.1) followed by immunoprecipitation of BiP (**b**, **d**, **f**, **h**) or Hsp70 (**c**, **e**, **g**, **j**) and western blot detection of E protein and BiP or Hsp70 in lysates and precipitates. BiP and Hsp70 were detected with a specific anti-BiP or one of two different anti-Hsp70 antibodies, TBEV and LGTV E with anti-LGTV E antibody and WNV and USUV E with anti-WNV E antibody. Representative blots of at least three independent replicates are shown. Input lysates are shown as controls for co-immunoprecipitations.

To validate these interactions in the context of authentic viral infection, we infected CaCo-2 cells with TBEV *Neudoerfl* or LGTV *TP21* at an MOI of 0.1 for 48 hours, followed by BiP or Hsp70 specific pull-downs (Fig. 2a). We detected complex formation between BiP and Hsp70 with the authentic E proteins of both TBEV (Fig. 2b, c) and LGTV (Fig. 2d, e) during infection. We next examined whether these interactions extend to more distantly related mosquito-borne orthoflaviviruses by performing infection experiments with WNV *NY99* and Usutu virus (*Orthoflavivirus usutuense*, USUV) *HAN/SN/2018*. Similar to the tick-borne viruses, we observed BiP and Hsp70 interaction with the E proteins of WNV (Fig. 2f, g) and USUV (Fig. 2h, j). Since overexpression of viral proteins may potentially result in misfolding or mislocalization, the interactions reported here were validated using authentic viral proteins during infection, confirming that BiP engages the E protein under physiologically relevant conditions. These findings suggest that BiP and Hsp70 may play a conserved role in the orthoflavivirus life cycle through direct interactions with the viral E protein.

### BiP and Hsp70 interact with tick-borne orthoflavivirus NS1 proteins, but not with NS1 proteins of mosquito-borne orthoflaviviruses

**Fig. 3:**
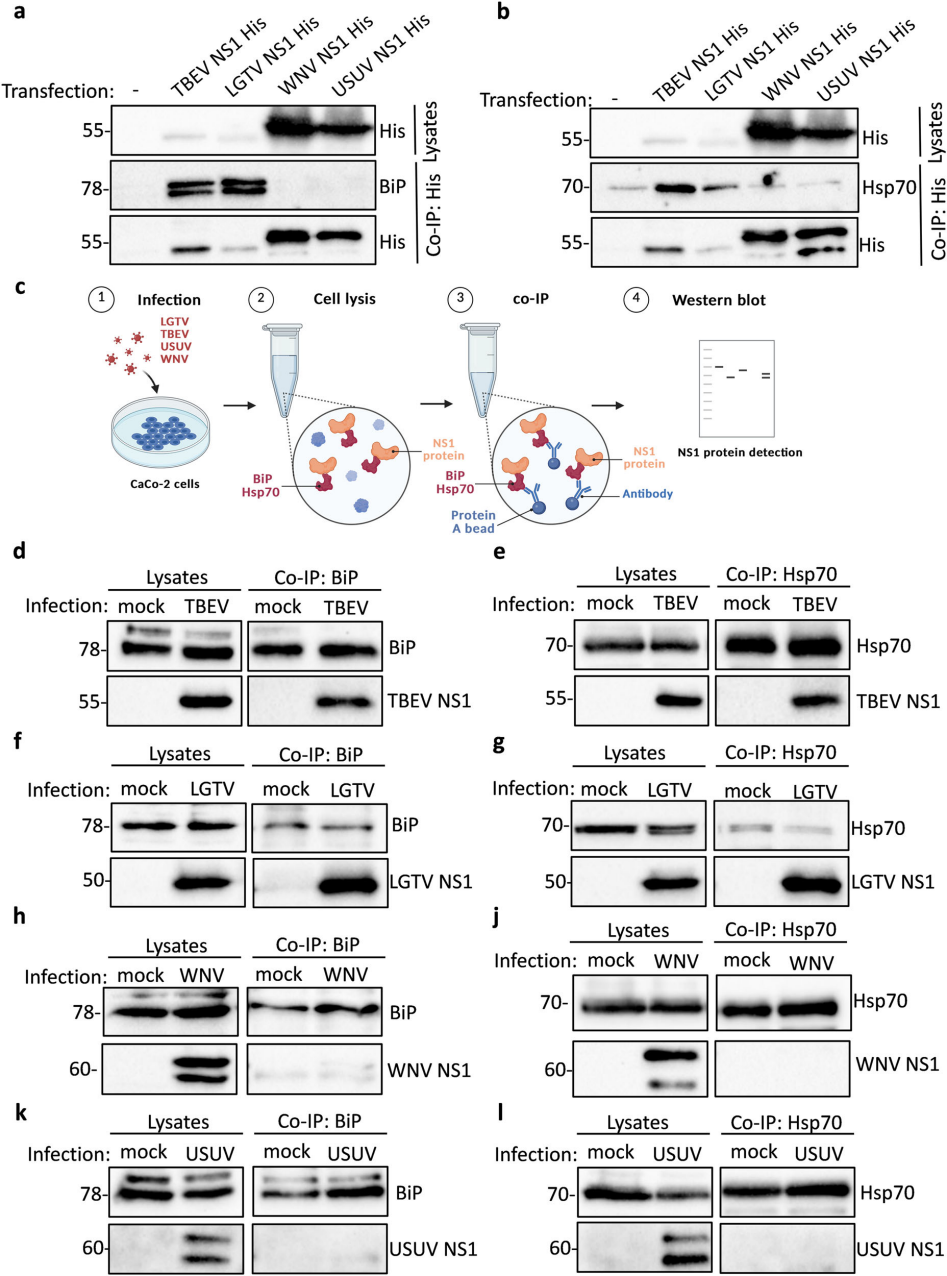
BiP and Hsp70 interact with NS1 proteins of TBEV and LGTV, but not with NS1 proteins of WNV and USUV. **a**, **b** Overexpression of His-tagged TBEV, LGTV, WNV, and USUV NS1 proteins in HEK293T cells followed by immunoprecipitation with anti-His antibody and western blot detection of BiP (**a**) or Hsp70 (**b**) and the respective viral proteins in lysates and precipitated samples. BiP and Hsp70 were detected with a specific anti-BiP or anti-Hsp70 antibody and His-tagged NS1 proteins with an anti-His antibody. Cells were transfected with empty vector as a negative control (-). Representative blots of at least three independent replicates. **c** Schematic representation of the experimental workflow for infection/immunoprecipitation experiments. **d–l** Caco-2 cell infection with TBEV (**d**, **e**), LGTV (**f**, **g**), WNV (**h**, **j**), or USUV (**k**, **l**) (MOI 0.1) for 48 h followed by immunoprecipitation of BiP (**d**, **f**, **h**, **k**) or Hsp70 (**e**, **g**, **j**, **l**) and western blot detection of NS1 protein and BiP or Hsp70 in lysates and precipitates. BiP and Hsp70 were detected with a specific anti-BiP or one of two anti-Hsp70 antibodies, TBEV and LGTV NS1 with anti-LGTV NS1 antibody and WNV and USUV NS1 with anti-WNV NS1 antibody. Mock infected cells were used as negative controls. Representative blots of at least three independent replicates are shown. Input lysates are shown as controls for co-immunoprecipitations.

**Fig. 4:**
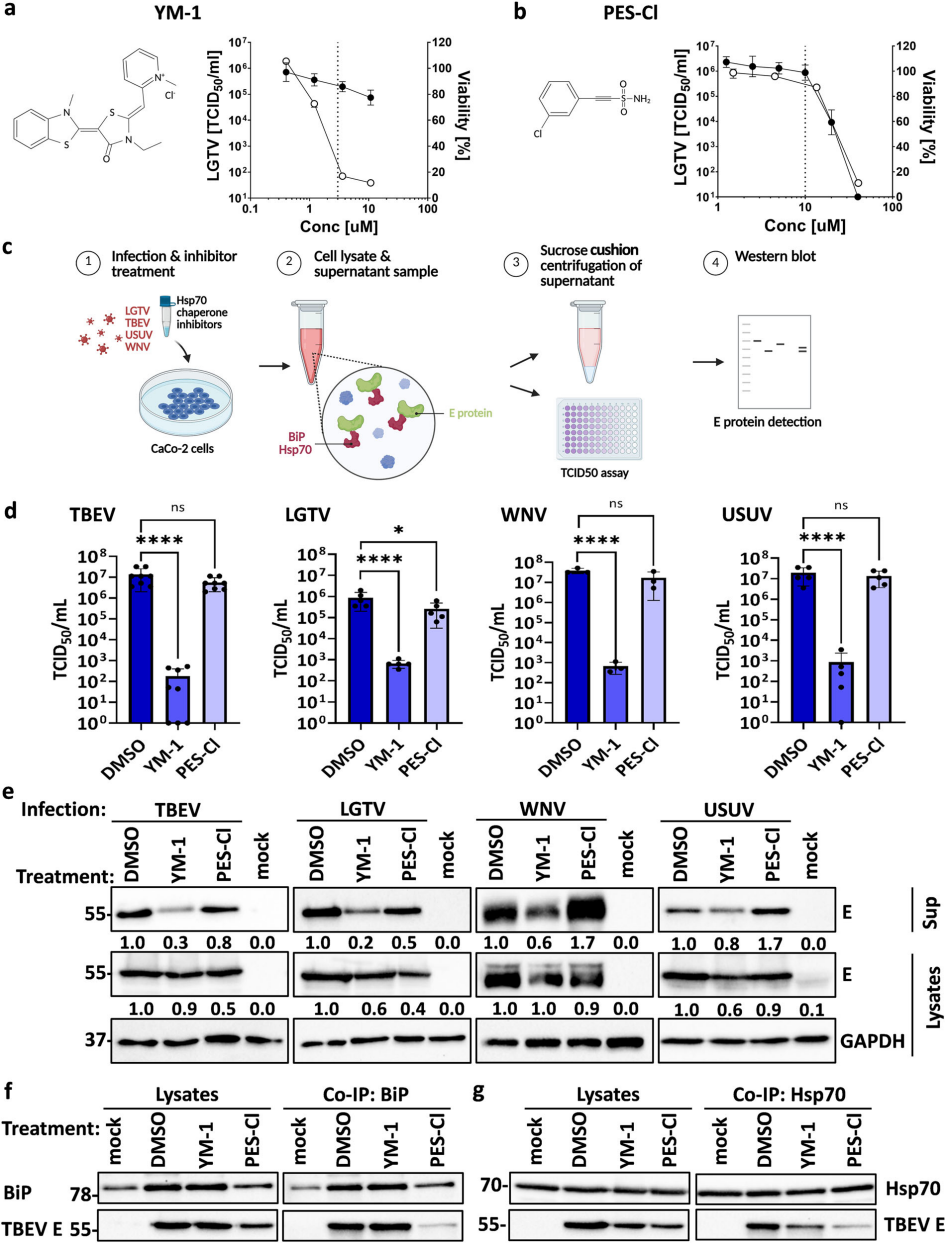
Chemical inhibition of BiP/Hsp70 by YM-1 significantly suppresses orthoflavivirus infectious particle release. **a**, **b** Structural formula and cytotoxicity test of Hsp70 chaperone inhibitors YM-1 (**a**) and PES-Cl (**b**). CaCo-2 cells were treated for 48 h with several inhibitor concentrations. Cell viability was determined in % normalized to DMSO control (solid circles, *n* = 3). In parallel, CaCo-2 cells were infected with LGTV (MOI 0.1) and treated for 48 h with several inhibitor concentrations. The infectious supernatants were then titrated on BHK-21 cells to determine TCID50/ml (open circles, *n* = 2). The inhibitor concentrations used in downstream experiments are indicated by dotted lines. **c** Schematic representation of the experimental workflow for infection/chaperone inhibitors experiments. **d**, **e** CaCo-2 cells were treated with YM-1 (3 µM) or PES-Cl (10 µM) and infected with TBEV, LGTV, WNV, or USUV (MOI 0.1) for 48 h. **d** Viral titers were determined in TCID_50_/mL. Data show mean ± SD of *n* = 8 (TBEV), *n* = 5 (LGTV, USUV), or *n* = 3 (WNV) from at least two independent experiments. Ordinary one-way ANOVA followed by Dunnett’s multiple comparisons test was performed for statistical analysis and asterisks indicate significant differences (ns *p* > 0.05; * *p* ≤ 0.05; **** *p* ≤ 0.0001). **e** Western blot detection of E protein in lysates and pelletized supernatants. TBEV E was detected with an anti-TBEV E antibody, LGTV E with anti-LGTV E antibody and WNV and USUV E with an anti-WNV E antibody. GAPDH was detected in lysates as loading control using an anti-GAPDH antibody. Representative blots of at least three independent replicates are shown. Band intensities of the representative blots were quantified using Bio-Rad Image Lab software and normalized to GAPDH; values are shown relative to DMSO control. **f**, **g** CaCo-2 cells were treated with YM-1 (3 µM) or PES-Cl (10 µM) and infected with TBEV (MOI 0.1) for 30 h followed by immunoprecipitation of (f) BiP or (g) Hsp70 and western blot detection of TBEV E protein and chaperones. A representative blot of two replicates is shown.

Given that BiP and Hsp70 interact with the E proteins of both tick- and mosquito-borne orthoflaviviruses, we next examined whether these chaperones also associate with NS1, another ER-localized viral glycoprotein involved in orthoflavivirus replication and immune evasion. To assess this, we used the overexpression system in HEK293T cells for the NS1 proteins of TBEV, LGTV, WNV, and USUV (Fig. 3a, b). To ensure proper co-translational translocation into the ER lumen, protein trafficking along the secretory pathway, and subsequent secretion of the NS1 proteins, the signal peptide of CD33 was added upstream of the protein-coding sequence in all NS1 expression constructs^38^. Following pull-down of the His-tagged NS1 proteins using an anti-His antibody, we found that NS1 from TBEV and LGTV formed complexes with both BiP and Hsp70 (Fig. 3a, b). In contrast, no or minimal interaction was detected between BiP or Hsp70 and the overexpressed NS1 proteins of WNV or USUV (Fig. 3a, b). To confirm these findings in the context of authentically processed NS1 proteins during viral infection, we infected CaCo-2 cells with TBEV, LGTV, WNV, or USUV (MOI 0.1) and performed BiP and Hsp70 pull-downs (Fig. 3c). Consistent with our overexpression results, we observed complex formation between BiP/Hsp70 and NS1 from TBEV (Fig. 3d, e) and LGTV (Fig. 3f, g), while no interaction was detected for WNV (Fig. 3h, j) or USUV NS1 (Fig. 3k, l). These findings reveal a striking difference between tick-borne and mosquito-borne orthoflaviviruses in their interactions with host ER chaperones. The overall levels of BiP and Hsp70 detected in LGTV-infected lysates were lower than in infections with other orthoflaviviruses. This likely reflects both lower replication efficiency of LGTV (see Fig. 4d) and correspondingly reduced induction of ER stress (as described by us and others^26,29,31,34,39^). Differences in antibody sensitivity may have contributed to differences in NS1 detection levels (Fig. 3f, g), as the anti-LGTV NS1 antibody may preferentially recognize LGTV NS1 compared to TBEV NS1. While both virus groups include BiP and Hsp70 in their E protein interactomes, only tick-borne viruses appear to specifically recruit these chaperones to their NS1 proteins. This suggests that NS1 proteins of tick-borne orthoflaviviruses may have distinct folding, trafficking, or chaperone-dependency compared to their mosquito-borne counterparts, potentially influencing viral replication or immune modulation.

### Chemical inhibition of BiP/Hsp70 by YM-1 significantly suppresses orthoflavivirus infectious particle release

To explore the functional role of BiP and Hsp70 in infection by the described orthoflaviviruses, we used two Hsp70 chaperone inhibitors: YM-1, a close derivative of MKT-077 that binds to the nucleotide-binding domain, locking the chaperone in the inactive ADP-bound state^40,41^, and 2-(3-chlorophenyl) ethynesulfonamide (PES-Cl), which binds the substrate-binding domain, directly interfering with protein interactions^42^. Although both compounds likely inhibit several members of the Hsp70 family with similar sensitivity, they affect Hsp70 function through distinct mechanisms^41–43^. Initially, we tested CaCo-2 cell viability and LGTV replication in the presence of a range of different concentrations of YM-1 (Fig. 4a) and PES-Cl (Fig. 4b). While a strong reduction of infectious LGTV titers was observed for YM-1 concentrations that maintained a greater than 80% viability (Fig. 4a), no effect on LGTV replication was observed in the non-toxic range of PES-Cl (Fig. 4b). The following experiments were therefore conducted with 3 µM YM-1 which for LGTV resulted in 2 × 10^2^ TCID50/ml infectivity and 87% viability of CaCo-2 cells, and 10 µM PES-Cl which resulted in 3×10^5^ TCID50/ml infectivity and 99% viability of CaCo-2 cells (Fig. 4a, b). CaCo-2 cells were then infected with TBEV *Neudoerfl*, LGTV *TP21*, WNV *NY99*, or USUV *HAN/SN/2018* at an MOI of 0.1 and treated with YM-1 (3 µM) and PES-Cl (10 µM) (Fig. 4c). We first assessed the effects on particle release and infectivity by quantifying viral titers (Fig. 4d) and measuring the amount of E protein in supernatants (Fig. 4e). YM-1 treatment significantly reduced viral infectivity (Fig. 4d). Similarly, YM-1 treatment significantly decreased E protein levels in the supernatants for all tested orthoflaviviruses, suggesting an effect on particle formation and/or release (Fig. 4e). In contrast, PES-Cl had no effect on E protein levels or infectivity compared to the control (Fig. 4d, e). Infection efficiencies were comparable across viruses, with LGTV showing slightly lower replication, as confirmed by TCID_50_ assay (Fig. 4d). The observed effects of YM-1 on infectious viral titers and particle release were consistent across TBEV, WNV, LGTV, and USUV infections. These findings suggest that YM-1 blocks the formation and release of infectious orthoflavivirus particles, possibly related to the complex formation between the chaperones and the orthoflavivirus E proteins. However, the lack of effect of PES-Cl on particle release and infectivity suggests that direct interaction with the substrate-binding domain of BiP or Hsp70 is either not required for their function during the orthoflavivirus life cycle or not effectively disrupted by PES-Cl in these experiments. To further assess the specificity of the interaction of BiP or Hsp70 with orthoflavivirus glycoproteins as demonstrated in Fig. 1, we performed co-immunoprecipitation in the presence of the Hsp70 substrate-binding domain inhibitor PES-Cl. Consistent with inhibition of direct chaperone–client binding, PES-Cl reduced the interaction between BiP or Hsp70 and TBEV E protein (Fig. 4f, g). In contrast, treatment with the nucleotide binding domain inhibitor YM-1 did not affect complex formation between the TBEV E protein and BiP or Hsp70.

### BiP/Hsp70 inhibition by PES-Cl blocks NS1 secretion in TBEV and LGTV infection, while WNV and USUV NS1 remain unaffected

**Fig. 5:**
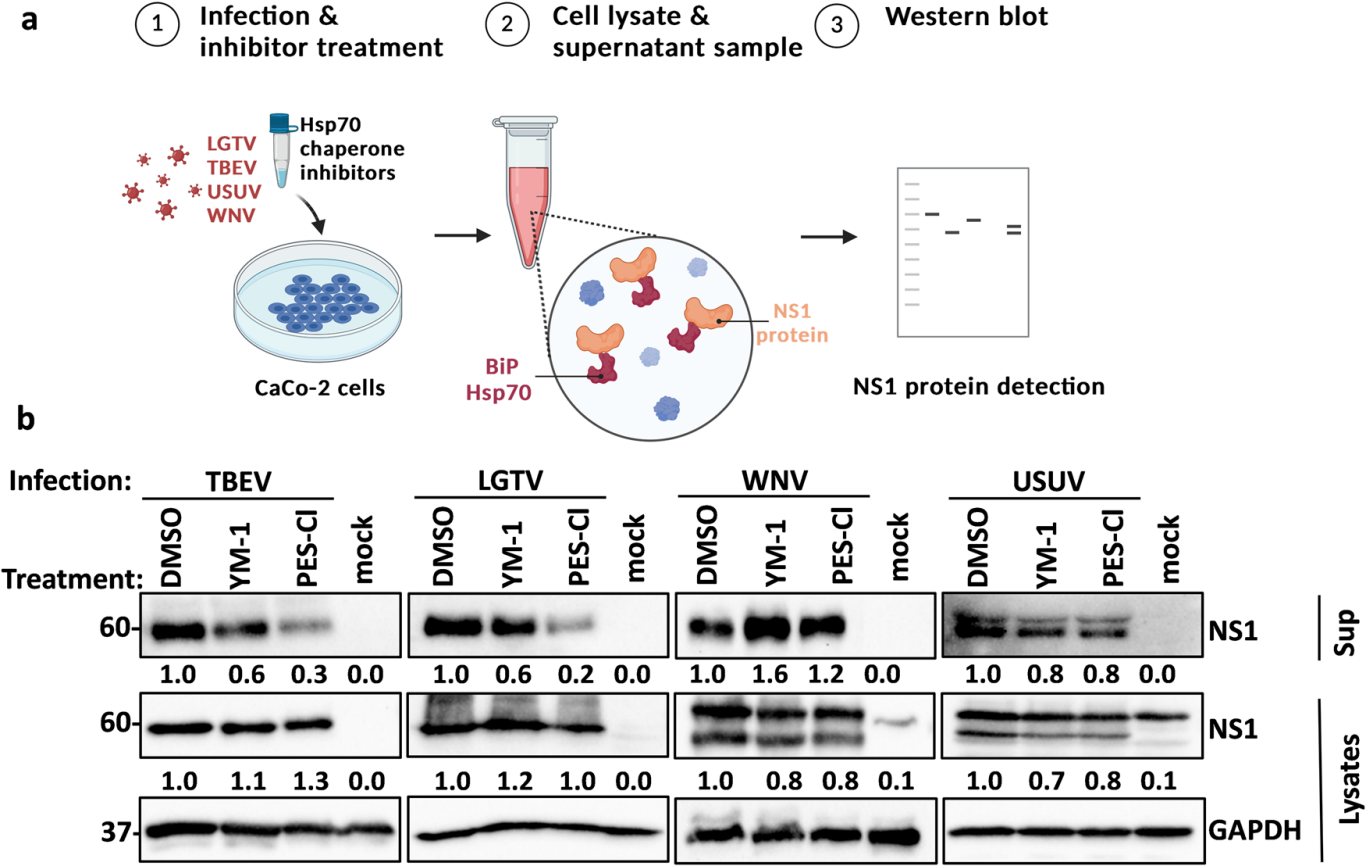
BiP/Hsp70 inhibition by PES-Cl blocks NS1 secretion in TBEV and LGTV infection, while WNV and USUV NS1 remain unaffected. **a** Schematic representation of the workflow for infection/chaperone inhibitors experiments. **b** CaCo-2 cells were treated with YM-1 (3 µM) or PES-Cl (10 µM) and infected with TBEV, LGTV, WNV, or USUV (MOI 0.1) for 48 h followed by western blot detection of NS1 protein in lysates and supernatants. TBEV and LGTV NS1 were detected with an anti-LGTV NS1 antibody and WNV and USUV NS1 with an anti-WNV NS1 antibody. GAPDH was detected in lysates as loading control using an anti-GAPDH antibody. Representative blots of at least three independent replicates are shown. Band intensities of the representative blots were quantified using Bio-Rad Image Lab software and normalized to GAPDH; values are shown relative to DMSO control.

To further investigate the role of Hsp70 chaperones in orthoflavivirus infection, we analyzed the expression and secretion of the NS1 protein, which serves several important functions during orthoflavivirus infection. Membrane-bound in the ER, NS1 is part of the replication complex, and when secreted, it plays a role in immune evasion and pathogenesis^12,24,44^. Following the experimental procedure described above, NS1 expression and secretion were assessed by western blotting. Notably, YM-1 treatment had no effect on NS1 secretion (Fig. 5b). In contrast, treatment with PES-Cl, which inhibits the substrate-binding domain of Hsp70 chaperones, led to a marked reduction in the secretion of TBEV and LGTV NS1 proteins (Fig. 5b). This effect was not observed with WNV or USUV, where NS1 protein expression and secretion remained unchanged under PES-Cl treatment (Fig. 5b). These results independently confirm the differences we observed in the direct interaction between the different NS1 proteins and BiP/Hsp70 (Fig. 3). Together, these findings suggest that BiP/Hsp70 play a distinct role in TBEV and LGTV infection, compared to WNV and USUV, likely through direct interaction between the respective NS1 proteins and the substrate-binding site of BiP/Hsp70.

### Silencing BiP activity results in reduced production of E and NS1 proteins in TBEV and WNV infection

**Fig. 6:**
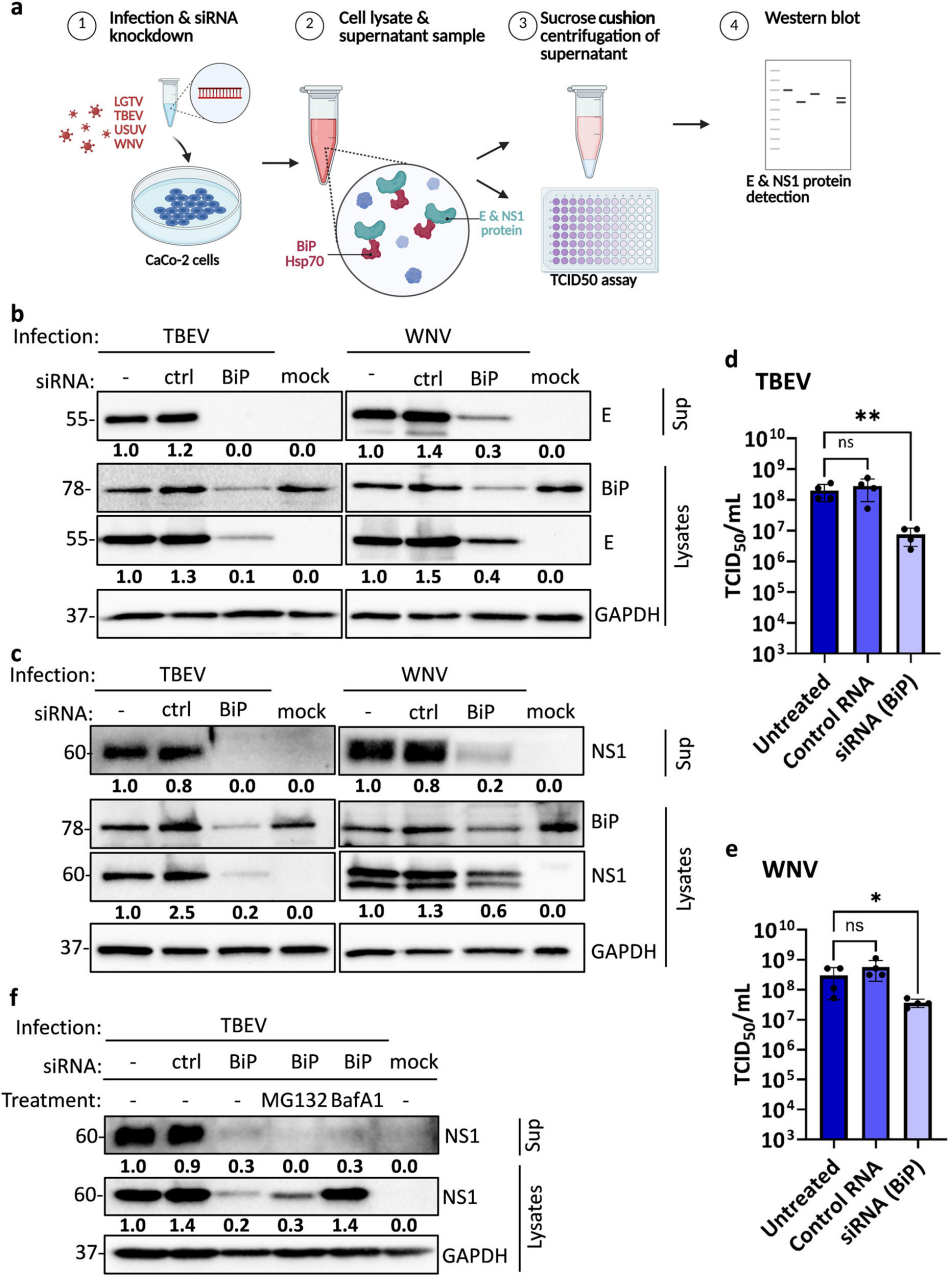
Silencing BiP by siRNA results in reduced production of E and NS1 proteins in TBEV and WNV infection. **a** Schematic representation of the workflow for infection/siRNA-mediated BiP knockdown experiments. **b–e** CaCo-2 cells were reverse transfected with control or BiP siRNA (40 pmol) for 48 h followed by infection with TBEV or WNV (MOI 0.1) for 48 h. Western blot detection of TBEV and WNV E (**b**) and NS1 (**c**) protein in lysates and supernatants. TBEV E was detected with an anti-TBEV E antibody, WNV E with anti-WNV E antibody, TBEV NS1 with anti-LGTV NS1 antibody and WNV NS1 with anti-WNV NS1 antibody. GAPDH was detected in lysates as loading control using an anti-GAPDH antibody. BiP was detected in lysates as knockdown control using an anti-BiP antibody. Representative blots of at least three independent replicates are shown. (d + e) Viral titers were determined for TBEV (**d**) and WNV (**e**) in TCID_50_/mL. Data show mean ± SD of n = 4 from at least two independent experiments. Ordinary one-way ANOVA followed by Dunnett’s multiple comparisons test was performed for statistical analysis and asterisks indicate significant differences (ns *p* > 0.05; * *p* ≤ 0.05; ** *p* ≤ 0.01). **f** Caco-2 cells were reverse-transfected with BiP siRNA as described above. Cells were treated with the proteasome inhibitor MG132 or the lysosomal inhibitor bafilomycin A1 (BafA1), and NS1 protein levels were analyzed by immunoblotting in cell lysates and culture supernatants. GAPDH was detected in lysates as loading control using an anti-GAPDH antibody. A representative blot of two replicates is shown. Band intensities of the representative blots were quantified using Bio-Rad Image Lab software and normalized to GAPDH; values are shown relative to untransfected/infected control.

Since YM-1 and PES-Cl likely target multiple members of the Hsp70 chaperone family with similar specificity, we performed a knockdown of BiP using a pool of four specific siRNAs to determine whether BiP is selectively required for orthoflavivirus particle formation and the secretion of TBEV and LGTV NS1 (Fig. 6a). First, we confirmed the efficacy of BiP silencing in CaCo-2 cells by measuring BiP mRNA levels via RT-qPCR and protein expression by western blot. Both approaches showed a significant reduction in BiP expression compared to the non-targeting siRNA control (ctrl) (Supplementary Fig. 1a, b). Next, CaCo-2 cells were infected with TBEV *Neudoerfl* and WNV *NY99* at MOI 0.1 and 48 hours post-siRNA transfection (Fig. 6a). In BiP-silenced cells, levels of both E and NS1 proteins in the lysates were substantially reduced compared to control siRNA-treated cells for both TBEV and LGTV (Fig. 6b, c). As a result, significantly less E and NS1 protein was detected in the supernatants (Fig. 6b, c). Interestingly, despite the reduced E protein levels in the supernatant, only a modest reduction in infectious viral titers was observed for both viruses (Fig. 6d, e). To investigate the fate of NS1 in BiP-silenced cells, we treated TBEV-infected cells with the proteasome inhibitor MG132 or the lysosomal inhibitor Bafilomycin A1 (BafA1). In cell lysates from BiP-deficient cells, NS1 expression was partially rescued by MG132 and fully restored by BafA1, indicating that NS1 can be degraded via both proteasomal and lysosomal pathways when BiP is absent (Fig. 6f). Importantly, neither inhibitor rescued NS1 secretion into the supernatant, suggesting that BiP is essential for NS1 secretion independent of its degradation. These results demonstrate that loss of BiP affects both NS1 stability and secretion, but the secretion defect cannot be attributed solely to enhanced degradation.

## Discussion

Hsp70 chaperones, including the ER-resident BiP, play essential roles in protein folding, quality control, and trafficking^45^. Our study provides direct evidence that orthoflavivirus envelope (E) proteins physically interact with both BiP and Hsp70, indicating these chaperones contribute to viral protein biosynthesis. Chaperone inhibitors have emerged as promising candidates for cancer therapy^46,47^, and several viruses dependent on cellular chaperones are inhibited by these compounds, with no evidence of resistant viral escape variants^48–50^. Chaperones are evolutionarily conserved across species, potentially representing a shared vulnerability that arboviruses must navigate when switching hosts. Inhibition of BiP/Hsp70 function with YM-1 disrupted E protein production and reduced infectious particle secretion, indicating that chaperone-mediated folding is critical for proper virion assembly. Virion assembly occurs at the ER membrane and is physically separated from the replication complex^51^. Viral prM and E proteins form the viral envelope and mediate budding of immature virions into the ER^51,52^. The immature virion travels via vesicular transport along the secretory pathway to the Golgi apparatus where mature virions are formed^51,53^. Thus, if the E protein is not folded correctly, for instance due to the lack of BiP/Hsp70 activity, envelope assembly is disrupted, potentially yielding incomplete or non-infectious virions.

As YM-1 locks Hsp70 chaperones in an ADP-bound state and prevents substrate cycling, our findings suggest that orthoflavivirus E protein requires active BiP/Hsp70 engagement for correct folding and membrane integration. This functional role aligns with prior reports of BiP involvement in dengue virus (DENV) virion assembly and its colocalization with Japanese encephalitis virus (JEV) E protein in infected cells^32,54^. While BiP is predominantly localized in the ER, it can translocate to the cell surface under stress and function as a viral receptor for orthoflavivirus E proteins, as demonstrated for ZIKV and JEV^36,55,56^. The dual role of Hsp70 chaperones in both intracellular folding and extracellular viral entry highlights their importance across multiple stages of the orthoflavivirus life cycle. Although YM-1 treatment resulted in detectable levels of E protein by western blot, detection does not guarantee functional fusogenic activity. Because BiP and other chaperones support proper folding and conformational maturation, the detected E protein may be structurally altered and impaired in function. The lack of detectable BiP binding to TBEV prM suggests that prM may fold efficiently in the ER or otherwise avoid exposure of hydrophobic regions that trigger BiP engagement. Antibody limitations prevented assessment of BiP/Hsp70 interactions with additional TBEV or LGTV proteins. However, pulldown experiments with PES-Cl support the specificity of BiP-E and Hsp70-E interactions, as SBD inhibition reduced binding. Differences in overall chaperone/viral protein levels across infections may reflect replication efficiency, ER stress, or antibody sensitivity. LGTV replication is lower than TBEV and follows delayed kinetics, which leads to reduced ER stress-mediated chaperone induction^34^. Together, these findings strengthen the conclusion that BiP and Hsp70 specifically engage the orthoflaviviral glycoproteins.

We also identified BiP/Hsp70 interaction with NS1 in certain orthoflaviviruses, revealing species-specific differences. TBEV and LGTV NS1 interacted with both BiP and Hsp70, whereas WNV and USUV did not. A possible explanation for this divergence may lie in the N-glycosylation profiles of the NS1 proteins. Distinct N-glycosylation sites (TBEV/LGTV: N85, N207; WNV/USUV: N130, N175, N207^57–61^) may influence protein folding and chaperone engagement within the ER^62^. While NS1 predominantly resides inside the ER where it has direct access to BiP, the mechanisms by which NS1 interacts with the mainly cytosolic Hsp70 are less obvious. Our pulldown experiments with the substrate-binding domain inhibitor PES-Cl show reduced binding of TBEV E protein to both Hsp70 and BiP, suggesting a direct interaction mediated by the substrate-binding domain. Hsp70 has also been reported to play an essential role in the life cycle of other orthoflaviviruses, such as DENV^36^, highlighting the functional relevance of this chaperone. Localization may involve ER-spanning replication complexes or membrane-associated interactions. While confocal microscopy could provide higher-resolution images of ER localization, co-localization alone does not prove direct protein interaction. Techniques, such as FRET, proximity ligation assays, or super-resolution microscopy could be applied to confirm direct binding.

Among Hsp70 inhibitors, only PES-Cl which binds the SBD and disrupts client interactions^42^ affected NS1. In TBEV/LGTV-infected cells, PES-Cl reduced NS1 secretion without affecting intracellular levels, suggesting impaired secretion rather than degradation. In contrast, PES-Cl had no effect on WNV/USUV NS1 secretion or stability. This indicates TBEV/LGTV NS1 requires Hsp70 chaperones for folding/trafficking, whereas WNV/USUV NS1 may use alternative pathways. This species-specific difference aligns with previous reports of BiP interacting with NS1 from other orthoflaviviruses. For instance, co-immunoprecipitation and co-localization studies demonstrated that DENV NS1 directly interacts with the substrate-binding domain of BiP in DENV-infected cells^63^. Proteomic analyses further support a functional interaction between DENV NS1 and BiP, showing that BiP is essential for proper NS1 folding and secretion^35^. Similar findings were reported for ZIKV NS1, where inhibition of BiP with the inhibitor HA15 impaired NS1 folding and secretion, highlighting a critical role for BiP in orthoflavivirus NS1 biogenesis^64,65^. Both DENV and ZIKV NS1 proteins contain two N-glycans (at N130 and N207) similar to TBEV and LGTV^66–69^. Our findings extend this model to TBEV and LGTV, suggesting that BiP-dependent folding of NS1 is a conserved feature among certain orthoflaviviruses but may not be universally required across all orthoflavivirus species.

Differences between YM-1 and PES-Cl inhibition suggest domain-specific consequences. YM-1 targets the NBD, preventing ATP hydrolysis and locking Hsp70 in a high-affinity substrate-bound state^40,41^. This effectively disrupts the chaperone’s ability to cycle between open and closed conformations, impairing its capacity to refold misfolded proteins and regulate proteostasis. In contrast, PES-Cl targets the SBD, reducing client interaction without blocking ATP cycling^42^. One possible explanation for the greater impact of YM-1 on viral infectivity is that blocking the ATPase function of Hsp70 chaperones has more profound effects on global cellular metabolism. Hsp70 chaperones regulate multiple aspects of protein homeostasis, including co-translational folding, degradation of misfolded proteins, and stress responses^45^. Inhibiting the NBD may not only interfere with viral protein folding but could also induce broader cellular stress responses, such as the UPR or autophagy, both of which can significantly influence viral replication^39,70,71^. If YM-1 leads to a global disruption of protein quality control, it may limit the cell’s ability to support viral replication more severely than PES-Cl. Another factor to consider is the potentially differential effects of these inhibitors on other Hsp70 family members. Cytosolic Hsp70 proteins are involved in various stages of viral infection, including capsid disassembly, genome trafficking, and immune evasion^36^. If YM-1 broadly disrupts these processes in addition to interfering with ER chaperones, this could explain its stronger effect on viral infectivity. YM-1 has been reported to affect other ATPases, including mitochondrial chaperones, which may compromise cellular energy homeostasis and indirectly impact viral replication^40,72^. Mitochondrial function plays a key role in antiviral signaling and metabolic adaptation during infection, and broad inhibition of ATPase activity could further exacerbate cellular stress^73,74^. PES-Cl, on the other hand, might be more selective for ER-resident chaperones, primarily impacting viral protein folding. Our results show that while BiP is essential for TBEV and LGTV NS1 protein secretion, disruption of BiP alone produced only modest reductions in viral replication. YM-1 reduced the titers of all tested orthoflaviviruses by several orders of magnitude, indicating that inhibition of BiP alone cannot account for its potent antiviral effect. Because YM-1 targets multiple Hsp70 family members, its antiviral activity may arise from inhibition of other chaperones that are more critical for orthoflavivirus infection. Importantly, in the absence of data identifying specific Hsp70 paralogs required for replication, we cannot exclude the possibility that YM-1’s strong antiviral effect stems from off-target activities outside the Hsp70 family.

Our experiments indicate that NS1 is subject to both proteasomal and lysosomal degradation in the absence of BiP. NS1 expression was partially restored by MG132 and fully by BafA1, but secretion remained blocked, highlighting BiP’s role in NS1 folding and export. These findings suggest that the reduced NS1 secretion observed upon BiP silencing is not merely a consequence of enhanced protein degradation but reflects a specific requirement for BiP in the NS1 maturation and export pathway. While siRNA-mediated BiP silencing reduced E and NS1 levels for all tested orthoflaviviruses, PES-Cl did not block NS1 secretion in WNV/USUV. It is currently unknown if PES-Cl sterically hinders interactions with all client proteins of Hsp70 chaperones with comparable efficacy or if differences in binding affinities modulate the effect. These differences could contribute to the discrepancies observed between the two approaches.

While CaCo-2 cells are not canonical neuronal targets of neurotropic orthoflaviviruses, TBEV can be transmitted via the alimentary route and enter through the intestine, making intestinal epithelial cells a relevant early target. CaCo-2 cells were chosen because they are well-characterized, permissive for orthoflavivirus replication, and suitable for the biochemical and inhibitor studies performed here. Nonetheless, findings in CaCo-2 cells should be confirmed in physiologically more relevant neuronal or endothelial cell types to fully capture virus-host interactions.

Overall, our findings highlight the importance of considering both domain-specific inhibition and broader cellular consequences when targeting chaperones for antiviral therapies. Understanding the roles of BiP and Hsp70 in viral protein synthesis and secretion is crucial for antiviral strategy development. Future studies comparing the global proteomic and transcriptomic changes induced by these inhibitors could provide further insight into their distinct effects on viral and host cell function. Directly targeting Hsp70 with YM-1 or PES-Cl mirrors cancer therapy approaches, but broad specificity requires careful consideration of host proteostasis.

## Methods

### Cell culture

Human embryonic kidney epithelial cells (HEK293T, ATCC CRL-3216), human intestinal epithelial cells (CaCo-2, ATCC HTB-37), human lung epithelial cells (A549, ATCC CCL-185), African green monkey kidney epithelial cells (Vero E6, ATCC CRL-1586) and Syrian golden hamster kidney fibroblasts (BHK-21, ATCC C-13) were cultured in high-glucose Dulbecco’s modified Eagle medium (DMEM) (Sigma-Aldrich, Taufkirchen, Germany) supplemented with 10% fetal bovine serum (FBS), 1% penicillin (10,000 U/mL)/streptomycin (10 mg/mL) solution and 2 mM L-glutamine solution (Sigma-Aldrich, Taufkirchen, Germany) at 37 °C with 5% CO_2_ in a humidified atmosphere. *Aedes albopictus* larvae cells (C6/36, ATCC CRL-1660) were cultivated in Schneiders’ Drosophila Medium (PAN Biotech GmbH, Aidenbach, Germany) supplemented with 10% FBS, 2 mM L-glutamine solution, 1% MEM NEAA (100×) without L-glutamine, 1 mM sodium pyruvate solution and 1% penicillin (10,000 U/mL)/streptomycin (10 mg/mL) (Sigma-Aldrich, Taufkirchen, Germany) at 28 °C. All cell lines were tested routinely for the existence of *Mycoplasma*.

### Virus strains

The tick-borne orthoflaviviruses TBEV strain *Neudoerfl* and Langat virus strain *TP21* were kindly provided by Gerhard Dobler. WNV strain *NY99* was a gift by Martin H. Groschup. USUV strain *HAN/SN/2018* was isolated from a great gray owl that had perished at Hannover zoo^75^. TBEV strain *Neudoerfl* (GenBank accession number U27495) was propagated in A549 cells. LGTV strain *TP21* (GenBank accession number AF253419) and WNV strain *NY99* (GenBank accession number KC407666.1) were propagated in Vero E6 cells. USUV strain *HAN/SN/2018* (GenBank accession number MT580899.1) was propagated in C6/36 cells. For infection experiments, CaCo-2 cells were seeded in 6-well plates or 10 cm dishes approximately 24 h before infection at a density of 2.5 × 10^5^ cells/mL. Before adding virus suspension to the cells, they were washed once with phosphate buffered saline (PBS) (Carl Roth, Karlsruhe, Germany). The cells were then infected with the respective orthoflavivirus strain at a multiplicity of infection (MOI) of 0.1 in serum-free medium for 1 h. Afterwards, the cells were washed again with PBS to remove unbound virus and incubated with DMEM containing 2% FBS for 48 h at 37 °C.

### Plasmid transfection

The sequences encoding the prM protein of TBEV *Neudoerfl* (GenBank: U27495.1, nucleotides 469-972) and the E proteins of TBEV *Neudoerfl* (GenBank: U27495.1, nucleotides 973-2460) and LGTV *TP21* (GenBank: AF253419.1, nucleotides 971-2458) were cloned into the mammalian expression vector pCAGGS via Gibson assembly. An N-terminal optimized signal sequence^37^ and a C-terminal polyhistidine tag (6x-His) were inserted into the vector. The sequences encoding different orthoflavivirus NS1 proteins (TBEV NS1: GenBank: U27495.1, nucleotides 2461-3516; LGTV NS1: GenBank: AF253419.1, nucleotides 2459-3514; WNV NS1: GenBank: DQ211652.1, nucleotides 2470-3525; USUV NS1: GenBank: MT580899.1, nucleotides 2475-3530) were cloned with an N-terminal CD33 signal sequence and a C-terminal 6x-His using the oligonucleotide primers listed in Supplementary Table 1^38^. The recombinant constructs were transformed into DH5-alpha competent Escherichia coli cells (New England Biolabs, Frankfurt, Germany) to produce plasmid DNA. The recombinant plasmids were transiently transfected into HEK293T cells using a calcium phosphate transfection protocol^76^.

### Antibodies for Immunoblotting

Primary antibodies used for immunoblotting are anti-6x-His tag monoclonal antibody (1:5000, clone HIS.H8, Thermo Fisher, Dreieich, Germany), anti-LGTV prM monoclonal antibody (1:5000, clone 13A10, obtained from the Joel M. Dalrymple - Clarence J. Peters USAMRIID Antibody Collection through BEI Resources, NIAID, NIH), anti-LGTV E monoclonal antibody (1:10000, clone 5G5, obtained from the Joel M. Dalrymple - Clarence J. Peters USAMRIID Antibody Collection through BEI Resources, NIAID, NIH), anti-LGTV NS1 monoclonal antibody (1:5000, clone 6E11, obtained from the Joel M. Dalrymple - Clarence J. Peters USAMRIID Antibody Collection through BEI Resources, NIAID, NIH), anti-TBEV E monoclonal antibody (1:5000, clone 19/1786, kindly provided by Matthias Niedrig^77^,), anti-GRP78 polyclonal antibody (1:5000, PA1-014A, Thermo Fisher, Dreieich, Germany), anti-Hsp70 antibody (1:2500, #4872, Cell Signaling, Frankfurt, Germany), anti-Hsp70 polyclonal antibody (1:5000, PA5-28003, Thermo Fisher, Dreieich, Germany), anti-flavivirus group antigen antibody (1:5000, clone D1-4G2-4-15, Sigma-Aldrich, Taufkirchen, Germany), anti-WNV E monoclonal antibody (1:2500, clone GT3029, GeneTex, Biozol, Eching, Germany), anti-WNV E polyclonal antibody (1:2500, GTX132052, GeneTex, Biozol, Eching, Germany), anti-WNV NS1 polyclonal antibody (1:2500, GTX132053, GeneTex, Biozol, Eching, Germany) and anti-GAPDH (1:5000, D16H11, Cell Signaling, Frankfurt, Germany). As secondary antibodies HRP-conjugated goat anti-mouse antibody (1:5000, clone Poly4053, BioLegend, Amsterdam, The Netherlands) and HRP-conjugated goat anti-rabbit antibody (1:5000, #7074, Cell Signaling, Frankfurt, Germany) were used.

### Protein analysis

Cell lysate and supernatant samples were collected 48 h post-transfection or post-infection (p.i.). For cell lysis, cells were washed with PBS and lysed in mild lysis buffer (150 mM NaCl, 0.1 mM Tris, 1% Triton X-100, pH 7.4, protease inhibitor cocktail 1:100 (Carl Roth, Karlsruhe, Germany)). Culture supernatants were centrifuged at 500 × g for 5 min to remove cells. To detect the viral particle-associated E protein, the collected supernatants were layered on a 20% sucrose cushion and centrifuged for 2 h at 4 °C at 17,000 × g. Most of the supernatant and sucrose was then removed until only a small pellet remained. For protein denaturation, 2 × loading buffer (0.125 M Tris pH 6.8, 4% SDS, 10% β-mercaptoethanol, 20% glycerol, and 0.5% bromophenol blue) was added to the samples, and they were boiled for 10 min at 95 °C. Proteins were separated by sodium dodecyl sulfate polyacrylamide gel electrophoresis (SDS-PAGE) and electroblotted to a nitrocellulose membrane (Carl Roth, Karlsruhe, Germany) using a semi-dry blotter (Carl Roth, Karlsruhe, Germany). Membranes were blocked in 5% low fat milk powder in Tris-buffered saline (TBS) containing 0.05% Tween-20 (TBS-T) for 1 h at room temperature (RT) and incubated with the indicated primary antibody overnight at 4 °C, followed by incubation with an HRP-conjugated secondary antibody for 1 h at RT. The chemiluminescent signal was generated using SuperSignal West Pico and SuperSignal West Femto Maximum Sensitivity substrates (Thermo Fisher, Dreieich, Germany) and developed using a ChemiDoc system (Bio-Rad, Munich, Germany).

### Co-immunoprecitation

For protein pull-down, 1 mL of cell lysates was incubated with 1 µl of the respective antibody while mixing overnight at 4 °C. The following day, 50 µl of Pierce™ Protein-A-Agarose beads (Thermo Fisher, Dreieich, Germany) were added to the sample and incubated for another 4 hours at 4 °C. After centrifugation for 2 min at 2,000 × g, the supernatant was aspirated and the beads were washed twice by adding 150 µl mild lysis buffer and repeated centrifugation. The samples were then mixed with 2 × loading buffer, boiled for 10 min at 95 °C and stored at -20 °C. Input lysates were included as controls for co-immunoprecipitation experiments and are presented alongside pulldown samples. As these experiments are intended as qualitative evidence of protein–protein interactions, no additional loading controls were included for input lysates.

### Hsp70 chaperone inhibitors

Two different small molecule inhibitors were used to inhibit the Hsp70 chaperone function. Cytotoxic effects of the Hsp70 chaperone inhibitors were tested using the CellTiter-Glo® 2.0 Cell Viability Assay kit (Promega, Walldorf, Germany). For this purpose, CaCo-2 cells were seeded at a density of 2.0 × 10^5^ cells/mL in white 96-well cell culture plates and treated the next day with the respective inhibitor in a variety of concentrations for 48 h. The readout was performed according to the manufacturer’s protocol. To test their effect on orthoflavivirus infection, the inhibitors were applied to the CaCo-2 cells during the medium change after virus inoculation and left on the cells for 48 hours. The Hsp70 nucleotide-domain inhibitor YM-1 (Sigma-Aldrich, Taufkirchen, Germany) was reconstituted in dimethyl sulphoxide (DMSO) at a stock concentration of 23.92 mM and applied to the cells at a final non-cytotoxic concentration of 3 µM. The Hsp70 substrate-binding site inhibitor PES-Cl (Sigma-Aldrich, Taufkirchen, Germany) was reconstituted in DMSO at a stock concentration of 463.7 mM and used at a final non-cytotoxic concentration of 10 µM. Cells were infected at a low multiplicity of infection (MOI 0.1) to allow measurement of viral protein production and release of infectious particles. Chaperone inhibitors (YM-1 or PES-Cl) were applied at the indicated concentrations, and supernatants and cell lysates were harvested at 48 hours post-infection. Earlier time points were not used because tick-borne flaviviruses, particularly LGTV, exhibit delayed replication kinetics compared to other flaviviruses. The 48-hour time point allowed consistent detection of viral antigens and quantification of infectious particle production. Lysates and supernatants were analyzed by western blot for expression of viral glycoproteins. For infection experiments, GAPDH was used as a loading control for lysates, and equal volumes of supernatants were analyzed, with lysates serving as internal controls. Total protein normalization of supernatants was not performed, as secreted viral proteins contribute to total protein content and normalization could confound the results. The collected supernatants were additionally titrated for infectious virus as described below.

### TCID_50_ assay

Viral infectious titers were quantified by TCID_50_ assay. Therefore, BHK-21 cells were seeded at a density of 1.5 × 10^5^ cells/mL in 96-well plates. The following day, infectious cell culture supernatants were titrated on the BHK-21 cells in 10-fold dilutions and the readout was performed by microscopic observation of cytopathic effects (CPE) 4 days p.i. For calculation of viral titers, the Reed & Muench protocol^78^ was used.

### siRNA-mediated knock-down of BiP

To knock down BiP activity, CaCo-2 cells were reverse transfected with ON-TARGETplus Human HSPA5 siRNA (Dharmacon, Horizon Discovery, Waterbeach, UK) and with ON-TARGETplus Non-targeting Control Pool (Dharmacon, Horizon Discovery, Waterbeach, UK) as a negative control. Therefore 20 pmol (12-well format) or 40 pmol (6-well format) of siRNA were mixed with 2 µl (12-well) or 4 µl (6-well) RNAiMAX reagent (Thermo Fisher, Dreieich, Germany) in 250 µl (12-well) or 500 µl Opti-MEM + GlutaMAX (Thermo Fisher, Dreieich, Germany). After an incubation time of 15 min at RT, the mix was added to the cell culture plates. CaCo-2 cells were seeded in antibiotic-free DMEM at a density of 2.0 × 10^5^ cells/mL on top of the siRNA mixture and incubated for 48 h at 37 °C. The cells were then either lysed to isolate the mRNA or infected with TBEV *Neudoerfl* or WNV *NY99* at MOI 0.1 as described above.

### Protein degradation inhibitors

Inhibitors of different protein degradation pathways were applied following siRNA-mediated BiP knockdown to assess the fate of orthoflavivirus proteins. Cytotoxic effects of the inhibitors were tested using the CellTiter-Glo® 2.0 Cell Viability Assay kit (Promega, Walldorf, Germany). For this purpose, CaCo-2 cells were seeded at a density of 2.0 × 10^5^ cells/mL in white 96-well cell culture plates and treated the next day with the respective inhibitor in a variety of concentrations for 16 h. The readout was performed according to the manufacturer’s protocol. To test their effect on orthoflavivirus protein levels, the inhibitors were applied to the CaCo-2 cells 32 h post infection and 16 h before sample collection. The proteasome inhibitor MG132 (Sigma-Aldrich, Taufkirchen, Germany) was reconstituted in DMSO at a stock concentration of 10 mM and applied to the cells at a final non-cytotoxic concentration of 10 µM. The lysosomal acidification inhibitor Bafilomycin A1 (Sigma-Aldrich, Taufkirchen, Germany) was reconstituted in DMSO at a stock concentration of 100 µM and used at a final non-cytotoxic concentration of 50 nM.

### BiP mRNA quantification by RT-qPCR

CaCo-2 cells were lysed using TRIzol reagent (Thermo Fisher, Dreieich, Germany) and total cellular RNA was isolated by using the Direct-zol RNA kit (Zymo Research, Freiburg, Germany) according to the manufacturer’s instructions. RT-qPCR was performed by using Luna Universal One-Step RT-qPCR Kit (New England Biolabs, Frankfurt, Germany) and preparing the samples according to the manufacturer’s protocol. BiP transcript level was quantified using specific primers (forward primer 5’-TGTTCAACCAATTATCAGCAAACTC-3’ and reverse primer 5’-TTCTGCTGTATCCTCTTCACCAGT-3’) (Eurofins Genomics Germany GmbH, Ebersberg, Germany) and normalized to GAPDH transcript levels (forward primer 5′-CCCATGTTCGTCATGGGTGT-3′ and reverse primer 5′-TGGTCATGAGTCCTTCCACGATA-3′) (Eurofins Genomics Germany GmbH, Ebersberg, Germany). The LightCycler 96 system (Roche Deutschland Holding GmbH, Grenzach-Wyhlen, Germany) was used for detection.

### Statistical analysis and illustrations

All statistical analysis were performed with GraphPad Prism 9.0 or 10.0 (GraphPad Software, San Diego, CA, USA). The data were analyzed for normal distribution using Shapiro-Wilk normality test. For parametric analyses, either unpaired two-tailed *t* test or ordinary one-way ANOVA followed by Dunnett’s multiple comparisons test were performed. A p-value of ≤ 0.05 was considered statistically significant, with the following significance levels: ns p > 0.05; * p ≤ 0.05; ** p ≤ 0.01; **** p ≤ 0.0001. BioRender (BioRender.com) was used to create schematic illustrations.

## Supplementary information

Supplementary data

## Data Availability

The data supporting this study are included in the article and its supplementary files. Original raw data, including uncropped blots and replicate experiments, are available from the corresponding author upon reasonable request. No large-scale datasets (e.g. sequencing or omics data) were generated in this study.

## Code availability

No code was generated or applied in the present study.
