# Supplementary material for: Divergent roles of Hsp70 chaperones in orthoflavivirus protein secretion and virion formation
Source: Npj Viruses. 2026 Feb 2;4:8. doi: 10.1038/s44298-026-00175-8 (PMC12864918; doi:10.1038/s44298-026-00175-8)
Supplement: Supplementary file 1 — Supplementary data [file 44298_2026_175_MOESM1_ESM.pdf]

Supplementary Material

Supplementary Table 1. Primer sequences used for HiFi assembly cloning of orthoflavivirus sequences into the pCAGGS expression vector. Heterologous signal sequences (optimized signal sequence optss for prM and E sequences and CD33 signal peptide CD33-SP for NS1 sequences) as well as 6xHis epitope tags are shown in bold and underlined. The sequence for LGTV E was amplified in two overlapping fragments.

Gene	Orientation	Primer (5' - 3')
**optss TBEV prM His**	Forward	AGCTCATCGATGCATGGTACC**ATGGTGGGCGTGGCCCTGGCCCTGGCCCTGGCCACCACCGCCATGGCC**ACGGTGAGGAAAGAAAGGG
	Reverse	AGGGAAAAAGATCTGCTAGCTCGAGTTA**ATGATGGTGGTGATGATG**AGCGTAAACCGGTGCCAAAC
**optss TBEV E His**	Forward	AGCTCATCGATGCATGGTACC**ATGGTGGGCGTGGCCCTGGCCCTGGCCCTGGCCACCACCGCCATGGCC**TCGCGTTGCACACACTTGG
	Reverse	AGGGAAAAAGATCTGCTAGCTCGAGTTA**ATGATGGTGGTGATGATG**CGCCCCCACTCCAAGGGT
**optss LGTV E His fragment 1**	Forward	AGCTCATCGATGCATGGTACC**ATGGTGGGCGTGGCCCTGGCCCTGGCCCTGGCCACCACCGCCATGGCC**TCGAGATGCACCCACCTG
	Reverse	TTTTCACTGCATGAGGCGTCCCAAACTC
**optss LGTV E His fragment 2**	Forward	GACGCCTCATGCAGTGAAAATGGATGTGTTCAATCTG
	Reverse	AGGGAAAAAGATCTGCTAGCTCGAGTTA**ATGATGGTGGTGATGATG**GGCTCCAACCCCCAGAGT
**CD33-SP TBEV NS1 His**	Forward	GTCTCATCATTTTGGCAAAGAATTC**ATGCCGCTGCTGCTACTGCTGCCCCTGCTGTGGGCAGGGGCCCTGGCT**GATGTTGGTTGCGCTGTG
	Reverse	GCTCGAGCATGCCCGGGTACCTTA**ATGATGGTGGTGATGATG**C
**CD33-SP LGTV NS1 His**	Forward	GTCTCATCATTTTGGCAAAGAATTC**ATGCCGCTGCTGCTACTGCTGCCCCTGCTGTGGGCAGGGGCCCTGGCT**GATGTGGGCTGCGCAGTG
	Reverse	GATCTGCTAGCTCGAGCATGCTTA**ATGATGGTGGTGATGATG**AGCC
**CD33-SP WNV NS1 His**	Forward	GTCTCATCATTTTGGCAAAGAATTC**ATGCCGCTGCTGCTACTGCTGCCCCTGCTGTGGGCAGGGGCCCTGGCT**GACACTGGGTGTGCCATAG
	Reverse	GATCTGCTAGCTCGAGCATGCTTA**ATGATGGTGGTGATGATG**AG
**CD33-SP USUV NS1 His**	Forward	GTCTCATCATTTTGGCAAAGAATTC**ATGCCGCTGCTGCTACTGCTGCCCCTGCTGTGGGCAGGGGCCCTGGCT**GACTCGGGATGTGCGATAG
	Reverse	GATCTGCTAGCTCGAGCATGCTTA**ATGATGGTGGTGATGATG**G

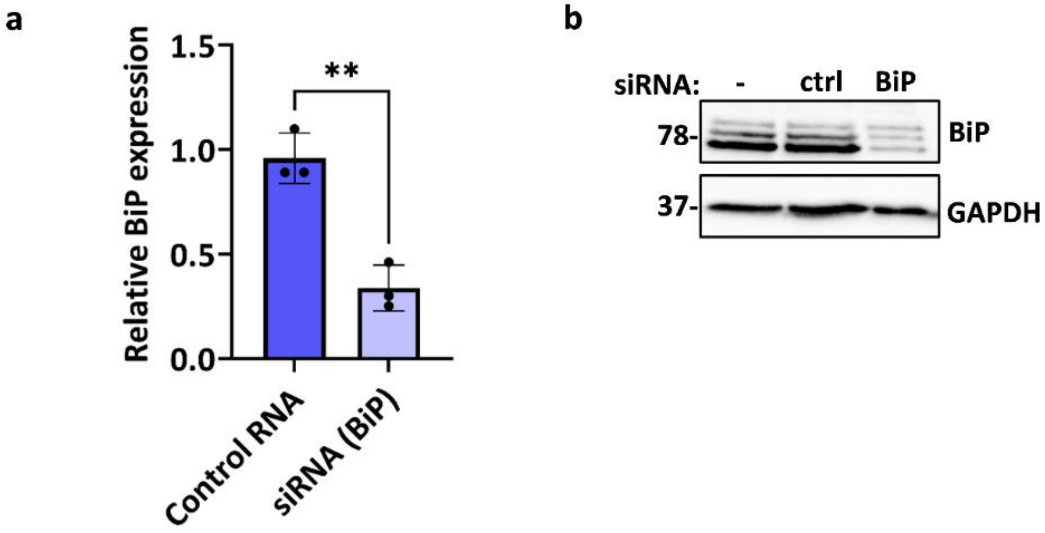

Supplementary Figure 1. BiP expression in CaCo-2 cells is reduced after BiP-specific siRNA treatment. (a+b) CaCo-2 cells were reverse transfected with control or BiP siRNA (20 pmol). Lysate samples were collected 48 h post-siRNA transfection. **(a)** Relative BiP expression was determined by quantifying BiP transcript level of cell lysates, normalized to GAPDH transcript level, using specific primers by RT-qPCR. Data show mean ± SD of n=3. Two-tailed unpaired t test was performed for statistical analysis and asterisks indicate significant differences (** $p \leq 0.01$). **(b)** Western blot detection of BiP using an anti-BiP antibody and GAPDH as loading control using an anti-GAPDH antibody in cell lysates. Representative blot of at least three independent replicates is shown.
